# The Composition and Structure of Biofilms Developed by *Propionibacterium acnes* Isolated from Cardiac Pacemaker Devices

**DOI:** 10.3389/fmicb.2018.00182

**Published:** 2018-02-14

**Authors:** Ken-ichi Okuda, Ryuichi Nagahori, Satomi Yamada, Shinya Sugimoto, Chikara Sato, Mari Sato, Tadayuki Iwase, Kazuhiro Hashimoto, Yoshimitsu Mizunoe

**Affiliations:** ^1^Department of Bacteriology, The Jikei University School of Medicine, Tokyo, Japan; ^2^Jikei Center for Biofilm Science and Technology, Tokyo, Japan; ^3^Department of Cardiac Surgery, The Jikei University School of Medicine, Tokyo, Japan; ^4^Biomedical Research Institute, National Institute of Advanced Industrial Science and Technology (AIST), Tsukuba, Japan

**Keywords:** biofilms, cell structure and function, extracellular matrix, *Propionibacterium acnes*, atmospheric scanning electron microscopy (ASEM), implanted devices

## Abstract

The present study aimed to understand the biofilm formation mechanism of *Propionibacterium acnes* by analyzing the components and structure of the biofilms. *P. acnes* strains were isolated from the surface of explanted cardiac pacemaker devices that exhibited no clinical signs of infection. Culture tests using a simple stamp culture method (pressing pacemakers against the surface of agar plates) revealed frequent *P. acnes* colonization on the surface of cardiac pacemaker devices. *P*. *acnes* was isolated from 7/31 devices, and the isolates were categorized by multilocus sequence typing into five different sequence types (STs): ST4 (JK18.2), ST53 (JK17.1), ST69 (JK12.2 and JK13.1), ST124 (JK5.3), ST125 (JK6.2), and unknown ST (JK19.3). An *in vitro* biofilm formation assay using microtiter plates demonstrated that 5/7 isolates formed biofilms. Inhibitory effects of DNase I and proteinase K on biofilm formation varied among isolates. In contrast, dispersin B showed no inhibitory activity against all isolates. Three-dimensional live/dead imaging of *P. acnes* biofilms with different biochemical properties using confocal laser microscopy demonstrated different distributions and proportions of living and dead cells. Additionally, it was suggested that extracellular DNA (eDNA) plays a role in the formation of biofilms containing living cells. Ultrastructural analysis of *P. acnes* biofilms using a transmission electron microscope and atmospheric scanning electron microscope revealed leakage of cytoplasmic components along with cell lysis and fibrous structures of eDNA connecting cells. In conclusion, the biochemical properties and structures of the biofilms differed among *P. acnes* isolates. These findings may provide clues for establishing countermeasures against biofilm-associated infection by *P. acnes*.

## Introduction

Biofilms are clusters of microorganisms formed on biotic or abiotic surfaces (Hall-Stoodley et al., 2004). According to a public announcement from the NIH, biofilm formation is estimated to be involved in over 80% of microbial infections^1^. Development of bacterial biofilms on indwelling medical devices, such as vascular catheters, prosthetic joints, and cardiac pacemakers, causes biofilm-associated infections (Percival et al., 2015). Bacterial cells in biofilms are embedded within a self-produced extracellular matrix (ECM) consisting of biomolecules such as nucleic acids, polysaccharides, and proteins (Flemming and Wingender, 2010). In biofilm of *Staphylococcus aureus* and *S. epidermidis*, extracellular DNA (eDNA), polysaccharide intercellular adhesion (PIA), and proteins have been shown to be components of ECM (Eckhart et al., 2007; Qin et al., 2007; Rice et al., 2007; Izano et al., 2008; Sugimoto et al., 2013, 2016; Chiba et al., 2015). The contribution of each component in biofilm formation is different among staphylococcal strains. Although the production of PIA is important in staphylococcal biofilm development, some strains form PIA-independent biofilms (O’Gara, 2007; Rohde et al., 2007). It is also known that profiles of ECM proteins are distinct among strains (Sugimoto et al., 2013). *Pseudomonas aeruginosa* produces at least three distinct alginate exopolysaccharides involving Pel and Psl that contribute to biofilm development and architecture (Ryder et al., 2007; Flemming and Wingender, 2010). Mucoid strains overproduce alginate and form uneven biofilms. Alginate is involved in the initial biofilm formation and is also responsible for the stability mature biofilms. In non-mucoid strains, which do not express alginate biosynthesis genes, Pel and Psl are involved in the establishment of biofilms (Flemming and Wingender, 2010). The secreted protein CdrA has been shown to bind directly to Psl and therefore constitutes a structural component of the *P. aeruginosa* biofilm matrix (Borlee et al., 2010). eDNA functions as an intercellular connector and plays a role in stabilization of the *P. aeruginosa* biofilm (Allesen-Holm et al., 2006; Yang et al., 2007). DNase I inhibits biofilm formation of *P. aeruginosa*, indicating that eDNA is required for the initial establishment of the biofilm (Whitchurch et al., 2002). Recently, it has been shown that *Haemophilus influenzae* produces an ECM composed of proteins, nucleic acids, and a β-glucan during biofilm formation. Additionally, eDNA appears to be an important component of ECM and essential in biofilm maintenance (Domenech et al., 2016).

Bacteria growing in biofilms are resistant to the host’s immune system and antibiotic therapy (Costerton et al., 1999; Melchior et al., 2006; Vlastarakos et al., 2007; Kania et al., 2008). Therefore, once a mature biofilm has developed, it can be extremely difficult to eradicate by conventional medical approaches, and invasive procedures such as removal of the infected device are required (Donlan, 2001). In the hospital, culture tests to identify pathogens are routinely performed on devices removed from patients with suspected infections, but not on those removed from patients not presenting any signs of infection. In general, devices in patients not presenting any signs of infection are assumed to be sterile, but several studies suggest that bacteria can colonize cardiac devices in asymptomatic patients (Pichlmaier et al., 2008; Kleemann et al., 2010; Rohacek et al., 2010). The role and clinical implication of bacterial colonization on such devices remain unclear. To better understand these phenomena, it is important to characterize the mechanism of device colonization.

Here, we investigated bacterial colonization on the surface of explanted cardiac pacemaker devices that exhibited no clinical signs of infection. As a result of culture tests followed by 16S rRNA sequencing, *Propionibacterium acnes* was isolated from 7/31 devices tested. *P. acnes* is an aerotolerant anaerobic Gram-positive commensal of the human skin, mouth, conjunctiva, and large intestine (Funke et al., 1997). *P*. *acnes* is usually responsible for late chronic infections and rarely causes acute infections related to medical devices (Levy et al., 2013). Culture tests using a simple stamp culture method that involved pressing pacemakers against the surface of agar plates indicated that *P. acnes* isolates formed biofilms on the surface of some pacemakers. Although there are several reports describing biofilm formation by *P*. *acnes in vivo* and *in vitro* (Ramage et al., 2003; Holmberg et al., 2009; Jahns et al., 2012), the exact mechanism is still unclear. In this study, we investigated the biochemical properties of *P*. *acnes* biofilms by analyzing ECM components and enzyme sensitivity of the biofilms. Furthermore, the fine structure of the *P. acnes* biofilm was observed using confocal laser scanning microscopy (CLSM) and atmospheric scanning electron microscopy (ASEM) (Nishiyama et al., 2010, 2014).

## Materials and Methods

### Culture Test of Removed Devices

Cardiac pacemaker devices were removed in the operation room, placed on sterilized steel containers, and stamped on Anaero Columbia Agar with Rabbit Blood (Becton Dickinson, Franklin Lakes, NJ, United States) containing 2.5% rabbit blood. They were subsequently incubated anaerobically in an air-tight container with an Anaero-Pack (Mitsubishi Gas Chemical Co., Inc., Tokyo, Japan) at 37°C for 7 days. Single-colony isolates were cultured anaerobically in Gifu Anaerobic Media (GAM) Broth (Nissui Pharmaceutical Co., Ltd., Tokyo, Japan) at 37°C for 3 days and stocked at -80°C.

### 16S rRNA Gene Sequencing

*Propionibacterium acnes* isolates were cultured in GAM Broth (Nissui Pharmaceutical Co., Ltd., Tokyo, Japan) in an air-tight container containing an Anaero-Pack (Mitsubishi Gas Chemical Co., Inc., Tokyo, Japan) at 37°C for 3 days. Cells were collected from 0.5 ml of cultures by centrifugation at 10,000 × *g* and 25°C for 10 min and suspended in an equal volume of double-distilled water (DDW). Then, the cell suspensions were incubated at 100°C for 10 min and supernatants containing genomic DNA (crude DNA extracts) were obtained after centrifugation at 10,000 × *g* and 25°C for 10 min. 16S rRNA genes of *P. acnes* isolates were amplified using the crude DNA extracts and universal gene primers 27f (5′-AGAGTTTGATCMTGGCTCAG-3′) and 1492r (5′-GGTTACCTTGTTACGACTT-3′) (Lane, 1991). PCR was performed in a total volume of 50 μl containing 1 ml of crude DNA extract, 1× PCR buffer, 0.4 mM dNTPs, 0.3 μM forward and reverse primers, and 1.0 U KOD FX Neo DNA polymerase (Toyobo Co., Ltd., Osaka, Japan). PCR reaction conditions were as follows: initial denaturation for 2 min at 94°C and 30 cycles consisting of 10 s at 98°C, 30 s at 53°C, and 1.5 min at 68°C. The amplified product was purified using the QIAquick PCR Purification Kit (Qiagen, Valencia, CA, United States) following the manufacturer’s protocol. Database searches for the generated DNA sequences were performed using the BLAST program of the National Center for Biotechnology Information^2^.

### Multilocus Sequence Typing

Sequence types (STs) and clonal complexes (CCs) of *P. acnes* isolates were determined following the method reported by McDowell et al. (2012). Briefly, PCRs were performed using the extracted genomic DNA samples and primer sets targeting *aroE, atpD, guaA, lepA, sodA, gmk, tly*, and *camp2*. DNA sequences of the amplified fragments were analyzed using the *P. acnes* Multilocus Sequence Typing (MLST) Databases^3^ to determine STs and CCs.

### Biofilm Formation and Measurement

*Propionibacterium acnes* isolates were cultured anaerobically in GAM Broth (Nissui Pharmaceutical Co., Ltd., Tokyo, Japan) in an air-tight container containing an Anaero-Pack (Mitsubishi Gas Chemical Co., Inc., Tokyo, Japan) at 37°C for 3 days. Cultures were diluted to an OD_595_ of 0.01 (corresponding to 2 × 10^7^ colony-forming units/ml) with fresh GAM Broth (Nissui Pharmaceutical Co., Ltd., Tokyo, Japan) supplemented with 1% glucose (GAMG Broth). Aliquots (200 μl) of this bacterial suspension were added to the wells of a 96-well flat-bottomed polystyrene plate and incubated anaerobically for 3 days at 37°C. Cell growth of *P. acnes* isolates was evaluated by measuring the absorbance at 595 nm. After removal of the supernatants, biofilms formed on the bottom of the wells were stained with 0.1% crystal violet for 10 min and subsequently washed twice with 200 μl of phosphate-buffered saline (PBS). Next, 200 μl of ethanol was added to the wells to extract crystal violet and the absorbance at 595 nm was measured using an Infinite 200 PRO Microplate Reader (Tecan, Mannedorf, Switzerland).

### Effect of Enzymes on Biofilm Formation

*Propionibacterium acnes* biofilms were formed and measured as described above except that 100 units/ml DNase I (Roche, Indianapolis, IN, United States), 100 μg/ml proteinase K (Sigma–Aldrich, St. Louis, MO, United States), or 20 μg/ml dispersin B (Kane Biotech Inc., Winnipeg, MB, Canada) (final concentrations) was added to the culture medium prior to biofilm formation. To eliminate the influence of the storage buffer components on biofilm formation, the storage buffers of DNase I and dispersin B were exchanged for DDW by repeated ultrafiltration with an Amicon Ultra 10K (Merck Millipore, Cork, Ireland) before use. Proteinase K powder was dissolved in DDW directly.

### ECM Extraction

Extracellular matrix was extracted based on previously described methods (Chiba et al., 2015). Biofilm cells, cultured anaerobically at 37°C for 3 days in GAMG Broth (2 ml), were harvested by centrifugation at 5,000 × *g* and 25°C for 10 min. After washing with PBS, the cells were suspended in the extraction buffer consisting of Tris–HCl (pH 8.0) and 1.5 M NaCl (5 μl of extraction buffer against 1 mg of wet cell weight), and incubated at 25°C for 30 min with agitation. Then, the cells were removed by centrifugation at 5,000 × *g* and 25°C for 10 min, and the extracted ECM in the supernatant was stored at -20°C until use.

### Quantification of ECM Components

The amount of DNA and proteins in the ECM was measured using a Qubit 3.0 Fluorometer (Thermo Fisher Scientific, Waltham, MA, United States) after staining with Qubit dsDNA HS Assay Kit (Thermo Fisher Scientific, Waltham, MA, United States) and Qubit Protein Assay Kit (Thermo Fisher Scientific, Waltham, MA, United States), respectively, according to the manufacturer’s instructions. *N*-acetyl glucosamine (GlcNAc) was detected by dot-blot analysis using wheat germ agglutinin conjugated to horseradish peroxidase (Goldstein and Hayes, 1978).

### Protein Identification

The extracted ECM was subjected to SDS–PAGE; subsequently, the gels were stained using the Silver Stain MS Kit (Wako, Tokyo, Japan). Protein bands of interest were excised from the gels. In-gel enzymatic digestion and protein identification by nanoLC-MS/MS were performed by Japan Proteomics Co., Ltd. (Sendai, Japan).

### CLSM Imaging

Biofilms of *P. acnes* JK12.2 and JK17.1 were formed after anaerobic cultivation in 500 μl of GAMG Broth using an 8-well chamber slide (Watson, Tokyo, Japan) in an air-tight container containing an Anaero-Pack (Mitsubishi Gas Chemical Co., Inc., Tokyo, Japan) at 37°C for 3 days. After removal of the supernatants, the biofilms formed on a slide glass were stained with 200 μl of Film-Tracer Live/Dead staining (Thermo Fisher Scientific, Waltham, MA, United States) according to the manufacturer’s protocol. The stained biofilm was washed once with 200 μl of DDW and observed with an LSM880 microscope (Carl Zeiss, Jena, Germany).

### Transmission Electron Microscopy Imaging

A biofilm of *P. acnes* JK12.2 was formed after anaerobic cultivation in 2 ml of GAMG Broth using a 12-well flat-bottomed polystyrene plate in an air-tight container containing an Anaero-Pack (Mitsubishi Gas Chemical Co., Inc., Tokyo, Japan) at 37°C for 3 days. Biofilms were fixed with 2.5% glutaraldehyde in 0.1 M phosphate buffer (pH 7.4) at room temperature and then with 1% osmic acid in phosphate buffer at 4°C for 1 h. Preparation of thin sections, staining, and microscopic observation were performed as described previously (Sugimoto et al., 2016).

### ASEM Imaging

A high-resolution image of *P. acnes* was obtained using the ClairScope ASEM System (JASM-6200, JEOL Ltd., Tokyo, Japan). *P*. *acnes* was cultured anaerobically in 2 ml of GAMG Broth using the standard 35-mm bio-ASEM dish in an air-tight container containing an Anaero-Pack (Mitsubishi Gas Chemical Co., Inc., Tokyo, Japan) at 37°C for 1 day. For heavy metal staining, the biofilms on the ASEM dish (JEOL Ltd., Tokyo, Japan) were fixed with 4% paraformaldehyde and 1% glutaraldehyde for 10 min, and subsequently stained using a slight modification of the National Center for Microscopy and Imaging Research (NCMIR) method developed by the Ellisman group (Deerinck et al., 2010) as recently reported (Memtily et al., 2015). For immuno-labeling, cells fixed with 4% paraformaldehyde were incubated first with a mouse anti-dsDNA monoclonal antibody (Abcam, Cambridge, MA, United States) and then with Fab fragments of goat anti-mouse IgG conjugated with 10-nm colloidal gold and Alexa Fluor 488 (Nanoprobes, Stony Brook, NY, United States) as described previously (Sugimoto et al., 2016). As a negative control for eDNA labeling, normal mouse IgG was used as a primary antibody. To tag the cells after immuno-labeling, positively charged 1.4-nm Nanogold particles (Nanoprobes, Stony Brook, NY, United States) were used as described previously (Sugimoto et al., 2016). In all samples, biofilms were immersed in 10 mg/ml D-glucose prior to ASEM observation, and imaged by ASEM at an acceleration voltage of 20 kV using backscattered electrons. The electron dose was less than 1.3 e^-^/Å^2^, which is 3% of the dose permitted in low-dose cryo-EM aiming at atomic-resolution single-particle reconstructions.

### Statistical Analysis

One-way ANOVA was performed to compare differences in mean values. Tukey’s multiple comparison test was applied when there was a significant difference in a comparison of groups. Statistical analyses were performed using Graph Pad Prism 6 software (GraphPad Software, La Jolla, CA, United States).

### Ethics Statement

In this study, we used pacemaker devices removed from patients for replacement. Culture tests of the pacemakers were performed after obtaining consent from each patient. This study was approved by the Ethics Committee of The Jikei University School of Medicine (No. 21-104 5682).

## Results

### *P*. *acnes* Colonization on Cardiac Pacemaker Devices

To examine bacterial colonization on the surface of cardiac pacemaker devices removed from patients with no symptoms of infection, culture tests were performed. After pressing pacemakers on blood-agar plates and cultivating them for 7 days under anaerobic conditions, 8/31 devices showed culture-positive results, defined as colony formation within the area that contacted the pacemaker surface. In some cases, numerous colonies were formed, indicating that bacterial cells adhere to all over the surface of the pacemakers (Supplementary Figure S1). To identify the strains colonizing the pacemakers, 16S rDNA sequencing targeting the V1–V9 region and BLAST database searches were performed. *P*. *acnes* was isolated from 7/31 devices (23% of total examined pacemakers and 88% of culture-positive pacemakers) (**Table 1**), in line with a report by Rohacek et al. (2010) on the high frequency of *P*. *acnes* on removed pacemakers. We also identified *P. granulosum* on PM13, indicating that at least two different species of bacteria seemed to colonize this pacemaker. Finally, *Staphylococcus hominis*, a coagulase-negative staphylococcus (CNS), was isolated from the remaining culture-positive pacemaker. However, as only one colony could be detected (data not shown), we assumed that it might correspond to contamination from the patient’s skin. To determine an association between certain lineages and characteristics of the *P*. *acnes* isolates, sequence typing was performed using the MLST scheme described by McDowell et al. (2012). As shown in **Table 2**, *P. acnes* isolates were categorized into five different STs. We could not determine the ST of JK19.3 by MLST analysis because of the failure of PCR amplification (data not shown). JK5.3 (ST124) and JK6.2 (ST125) shared 7/8 alleles, and both strains belonged to CC2 (**Table 2**). Only a single-nucleotide difference was found at nucleotide position 444 of the *lepA* gene fragment of JK5.3 and JK6.2 (*P. acnes* MLST Databases^4^), indicating that they are closely related strains.

**Table 1:** Bacterial identification by 16S rRNA sequencing.

Isolate	Origin	Accession	Best match (Accession)	Identity (%)
JK5.3	PM5	LC341274	*Propionibacterium acnes* TypeIA2 P.acn33 (CP003195)	100
JK6.2	PM6	LC341275	*Propionibacterium acnes* TypeIA2 P.acn33 (CP003195)	100
JK12.2	PM12	LC341276	*Propionibacterium acnes* ATCC 11828 (CP003084)	100
JK13.1	PM13	LC341277	*Propionibacterium acnes* ATCC 11828 (CP003084)	100
JK13.3	PM13	LC341278	*Propionibacterium granulosum* DSM 20700 (KF906605)	100
JK17.1	PM17	LC341279	*Propionibacterium acnes* KPA171202 strain KPA171202, complete genome (AE017283)	100
JK18.2	PM18	LC341280	*Propionibacterium acnes* KPA171202 strain KPA171202, complete genome (AE017283)	100
JK19.3	PM19	LC341281	*Propionibacterium acnes* strain L340 clone 1 (GQ496494)	100

**Table 2:** Multilocus sequence typing (MLST) analysis of *P. acnes* isolates.

Isolate	Phylotype	MLST-profile	ST	CC
JK5.3	IA2	1-1-1-5-1-4-31-2	124	CC2
JK6.2	IA2	1-1-1-5-11-4-31-2	125	CC2
JK12.2	II	17-4-2-4-4-6-10-12	69	CC72
JK13.1	II	17-4-2-4-4-6-10-12	69	CC72
JK17.1	IB	1-1-9-4-1-4-8-6	53	CC5
JK18.2	IA1	1-1-1-3-1-1-8-6	4	CC4
JK19.3	Unknown	–	–	–

*ST, sequence type; CC, clonal complex.*

### *In Vitro* Biofilm Formation Assay of *P*. *acnes* Isolates

**FIGURE 1:**
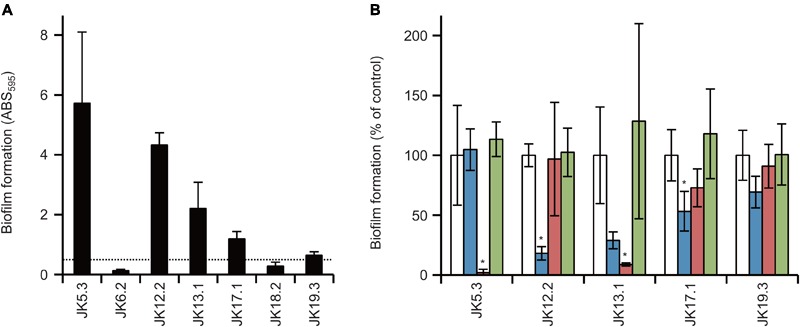
Biofilm formation by *Propionibacterium acnes* isolates and biofilm inhibition by enzymes. **(A)** Biofilm formation by *P. acnes* isolates cultured in GAMG broth, using 96-well plates and quantified by measuring ABS_595_. The dotted line represents the arbitrarily set threshold value (ABS_595_ = 0.5). **(B)** Enzymatic inhibition of biofilm formation using DNase I (blue), proteinase K (red), and dispersin B (green). No-enzyme controls (white) were set to 100 and relative values are shown. Means and standard deviations represent two independent experiments performed in triplicate. ^∗^*p* < 0.05 versus control.

We investigated the biofilm-forming capabilities of *P*. *acnes* isolates using a microtiter plate assay. No isolate formed biofilms in GAM Broth (data not shown). Among seven isolates, five isolates, i.e., all isolates except for JK6.2 and JK18.2, produced substantial biofilm (ABS_595_ > 0.5) in GAM Broth supplemented with 1% glucose (GAMG Broth) (**Figure 1A**). Interestingly, cell growth of JK5.3 (ST124) and JK6.2 (ST125) in GAMG Broth was nearly identical (data not shown), yet their biofilm-forming capacity was remarkably different. It seems that there are differences in the genes involved in biofilm formation between the two closely related strains belonging to CC2. Next, we investigated the efficacy of enzymes targeting *P*. *acnes* biofilm matrix constituents against biofilm formation by the five isolates. We used DNase I, proteinase K, and dispersin B, which digest DNA, protein, and poly-*N*-acetyl glucosamine (poly-GlcNAc), respectively. The enzymatic susceptibility of the biofilms varied among isolates (**Figure 1B**). DNase I significantly inhibited the biofilm formation by JK12.2 and JK17.1. In contrast, proteinase K significantly inhibited biofilm formation by JK5.3 and JK13.1. Dispersin B showed no inhibitory activity against all isolates. These results indicate that the contribution of DNA and proteins to biofilm formation differs among *P. acnes* isolates, and in some isolates, both molecules are involved in biofilm formation.

### ECM Components of *P. acnes*

**FIGURE 2:**
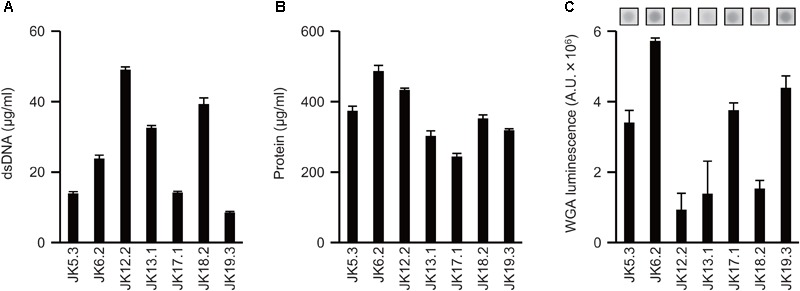
Extracellular matrix components of *P. acnes* biofilms. **(A–C)** Quantification of ECM components extracted from *P. acnes* biofilm cells: dsDNA **(A)**, protein **(B)**, and GlcNAc **(C)**. Photographs of dot-blot signals are shown in **(C)**. Means and standard deviations represent three independent experiments. A.U., arbitrary units.

Next, the ECM was extracted from *P*. *acnes* biofilms using the method developed by Chiba et al. (2015), and dsDNA, proteins, and GlcNAc in the ECM were quantified. The amount of each component varied in the tested strains (**Figure 2**) and did not necessarily correlate with the biofilm-forming capacity. For example, *P. acnes* JK6.2 presented the highest values for all ECM components except DNA, yet it formed very few biofilms. There was also no clear relationship between the amount of matrix components and enzymatic susceptibility of the biofilms. Abundance profiles of ECM components were similar among strains belonging to the same CC, such as JK5.3 and JK6.2 (CC2), and JK12.2 and JK13.1 (CC72), suggesting that CCs could be estimated by analyzing the composition ratio of matrix components.

### *P*. *acnes* ECM Protein Profiles

**FIGURE 3:**
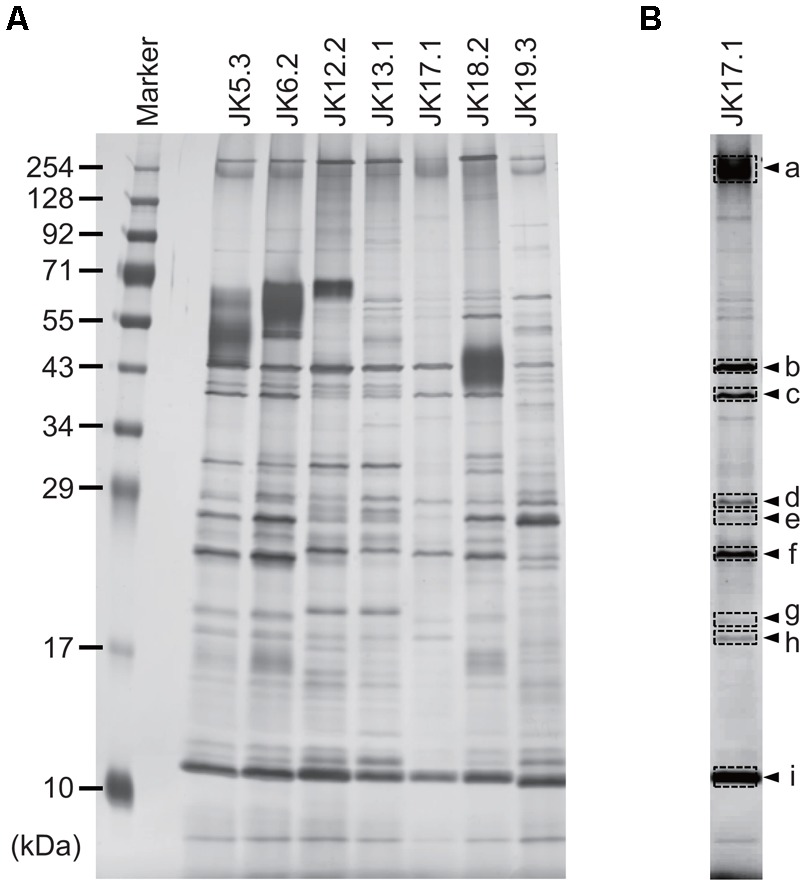
Profiles of proteins in ECMs. **(A)** Silver-stained gel showing proteins extracted from *P. acnes* ECMs and separated by SDS–PAGE. **(B)** The JK17.1 lane, following adjustment of brightness and contrast parameters. Arrowheads indicate bands analyzed by LC-MS/MS. Letters correspond to those in **Table 2**.

Proteins in *P. acnes* ECM were analyzed by SDS–PAGE and subsequent silver staining of the gel (**Figure 3A**). Band patterns were similar among strains belonging to the same CC, as in the case of JK5.3 and JK6.2 (CC2), or JK12.2 and JK13.1 (CC72). This finding suggests that isolates from the same CC produced similar ECM proteins. Next, we focused on the proteins in the ECM of *P. acnes* JK17.1, because it had the simplest ECM protein profile and biofilm formation by JK17.1 tended to be inhibited by proteinase K (**Figure 1B**). Proteins were extracted from bands separated by SDS–PAGE (**Figure 3B**), and analyzed by nano-LC-MS/MS. The protein identities are listed in **Table 3**. Some proteins were detected in multiple bands with different molecular weights, possibly as a result of aggregation or degradation. Christie–Atkins–Munch-Peterson (CAMP) factor 1 (CAMP1) was detected in bands a, e, and f. *P. acnes* CAMP1–5 represent potential secretory virulence factors, with homology to the CAMP factor of *Streptococcus agalactiae* (Jurgens et al., 1987; Lang and Palmer, 2003; Bruggemann, 2005). Nakatsuji et al. (2011) reported that *P. acnes* CAMP2 was cytotoxic to keratinocytes and macrophages. *P. acnes* isolates belonging to Type IB (phylotype of JK17.1) produce abundant CAMP1 (Valanne et al., 2005). Therefore, we speculated that secreted CAMP1 was associated with the cell surface and was found in the ECM. The functions of hydrolase and rare lipoprotein A (RlpA), two other proteins isolated from the ECM of *P. acnes* JK17.1, are still unknown. *P. acnes* hydrolase has a bacterial SH3 (SH3b) domain and NlpC/P60 cell wall hydrolase domains (Holland et al., 2010). Members of the NlpC/P60 family are known to be involved in cell wall hydrolysis and to cleave the linkage between D-Glu and diaminopimelic acid (or Lys) within benzylpenicillin stem peptides (Anantharaman and Aravind, 2003; Xu et al., 2015). RlpA, a widely conserved bacterial protein, was recently shown to possess lytic transglycosylase activity and to contribute to rod shape and daughter cell separation in *P. aeruginosa* (Jorgenson et al., 2014). Cytoplasmic proteins (enolase, 30S ribosomal protein S4, and DNA-binding protein HU) were also detected in the ECM; their presence is likely due to cell lysis releasing abundant cytoplasmic proteins.

**Table 3:** Proteins identified in the ECM of *P. acnes* JK17.1.

Sample	Protein	Molecular weight	Accession (gi)
a	CAMP factor	30,398	488472745
b	Enolase	45,530	488473032
c	Hydrolase	40,423	488482712
d	Hydrolase	40,423	488482712
e	CAMP factor	30,398	488472745
	30S ribosomal protein S4	23,229	488471162
f	CAMP factor	30,398	488472745
	Rare lipoprotein A	36,576	488472591
g	Rare lipoprotein A	52,306	50841310
h	Hydrolase	40,423	488482712
i	DNA-binding protein HU	9,588	488471011

### Three-Dimensional Structures of Biofilms and Distribution of Living/Dead Cells

**FIGURE 4:**
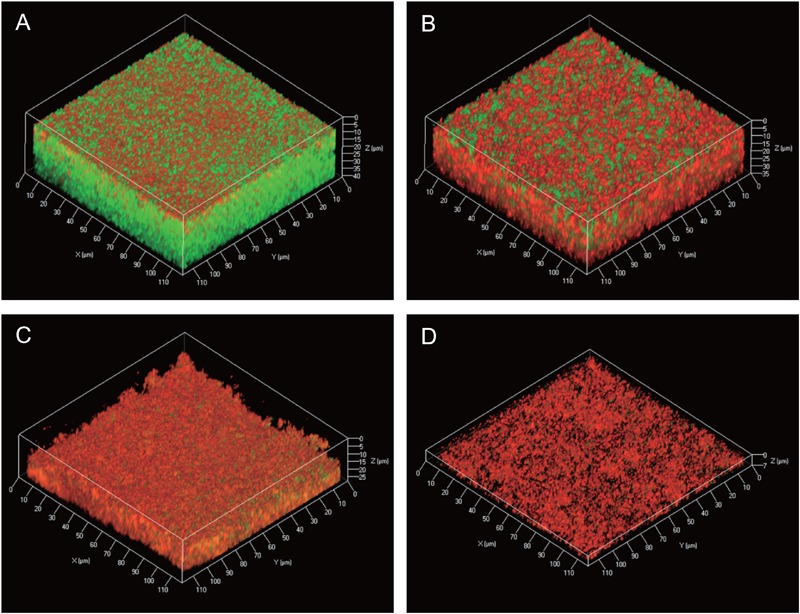
Three-dimensional live/dead imaging of *P. acnes* biofilms. **(A–D)** Three-dimensional images of a *P. acnes* JK12.2 biofilm cultured for 3 days were obtained by CLSM. Living and dead cells in the biofilm were stained by SYTO9 (green) and propidium iodide (red), respectively. Biofilm structures of JK12.2 **(A)**, JK17.1 **(B)**, JK12.2 cultured with DNase I **(C)**, and JK17.1 cultured with DNase I **(D)** are shown.

**FIGURE 5:**
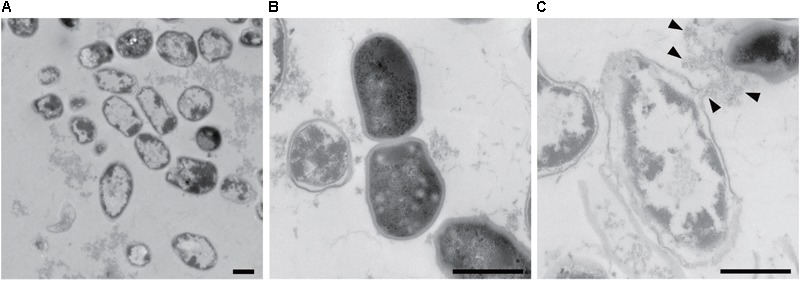
*Propionibacterium acnes* biofilm cells observed by TEM. Wide image **(A)**, enlarged image of a normal cell **(B)**, and enlarged image of a broken cell **(C)** are shown. Arrowheads indicate cytoplasmic components flowing out of the cells. Bars represent 0.5 μm.

Information about the 3D structures of *P. acnes* biofilms and the distribution and proportion of living/dead cells gives important insights to understand the mechanism of biofilm formation by *P. acnes*. Therefore, we observed live/dead stained biofilms of isolate JK12.2 and JK17.1, which belong to different STs, by CLSM. Both biofilms had smooth surfaces and a thickness of about 30 μm. In the biofilm of JK12.2, living cells (green) were the major cell population, and dead cells (red) were distributed on the top side. In contrast, in the biofilm of JK17.1, dead cells were widely distributed within the biofilm and constituted the majority of the cells (**Figures 4A,B** and Supplementary Figure S2). Planktonic cells were collected from biofilm cultures and subjected to live/dead staining; living cells constituted majorly of the cells in both isolates (Supplementary Figure S3). Therefore, it seems that cell death is actively induced on the surface of the JK12.2 biofilm and throughout the JK17.1 biofilm. Dead cells can be a supplier of ECM because the leakage of cytoplasmic components due to bacterial cell lysis has been shown to favor biofilm formation (Flemming and Wingender, 2010). To observe leakage of cytoplasmic molecules leaked from cells in the JK12.2 biofilm, we used transmission electron microscopy, which clearly showed an efflux of intracellular components from dead cells (**Figure 5**), possibly representing the release of ECM components during cell lysis. Next, we investigated the structures of biofilms formed in the presence of DNase I. In the presence of DNase I, the biofilm thickness (biomass) was significantly reduced in both JK12.2 and JK17.1 (**Figures 4C,D**), consisting with the results of the enzymatic inhibition experiment (**Figure 1B**). In contrast to the control, the biofilm of JK12.2 formed in the presence of DNase I had a rough surface and dead cells were the major cell population. Additionally, in the same condition, the biofilm of JK17.1 was thin and sparse and, similar to JK12.2, dead cells were the major cell population. We confirmed that DNA did not affect the viability of planktonic cells collected from biofilm cultures (Supplementary Figure S3). These results suggest that eDNA is involved in the formation of the 3D structures of *P. acnes* biofilms including living cells.

### Fine-Structural Observation of the Biofilm-Surface Interface

**FIGURE 6:**
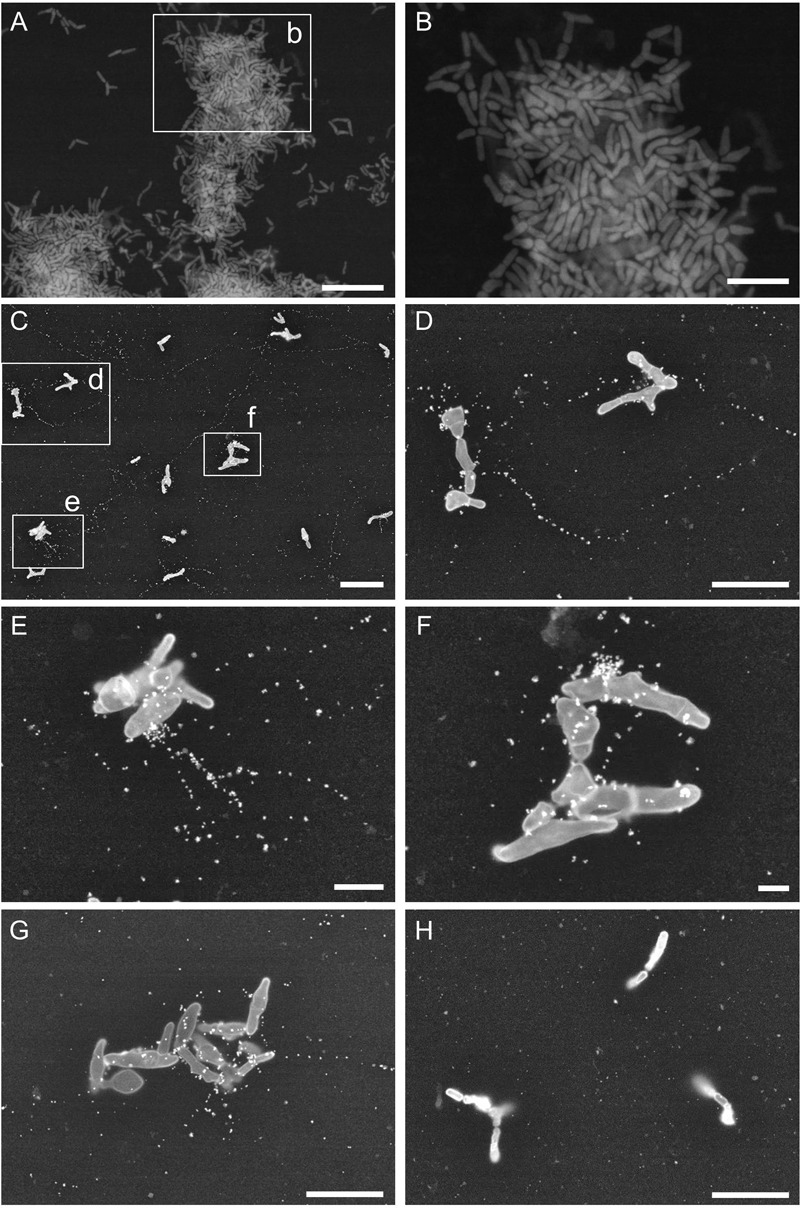
*Propionibacterium acnes* biofilm structures observed by ASEM. **(A,B)** Biofilm of *P. acnes* JK12.2 formed on an ASEM dish was stained with heavy metals using a modified NCMIR method. **(C–G)** Structures of eDNA visualized by immuno-ASEM. DNA molecules in the biofilm of *P. acnes* JK12.2 were immuno-labeled with mouse anti-dsDNA antibody and then with secondary antibody conjugated with 10-nm colloidal gold. After immuno-labeling, cells were counter-stained with positively charged 1.4-nm Nanogold. **(H)** As a negative control, the biofilm of *P. acnes* JK12.2 was labeled with normal mouse IgG and then with secondary antibody conjugated with 10-nm colloidal gold. Bars represent 10 μm (**A** and **C**), 5 μm (**B**, **D**, **G**, and **H**), 2 μm **(E)**, and 1 μm **(F)**.

Next, we used ASEM to obtain high-resolution images of eDNA localization in the biofilms. *P. acnes* JK12.2 was cultured in an ASEM dish. The ASEM dish is equipped with a SiN thin-film window in the center, allowing high-resolution imaging in liquids without destroying the natural structures of the biofilm (Sugimoto et al., 2016). After staining of the biofilm with heavy metals, clear images of the biofilm structures on the SiN film surface were obtained. A pleomorphic rod morphology, which is a characteristic of *P. acnes* cells, was observed (**Figures 6A,B**). Cells with diverse structures were arranged to fill the gap on the surface. Next, immuno-labeling of eDNA using an anti-dsDNA mouse IgG primary antibody and a 10-nm colloidal gold-conjugated anti-mouse IgG secondary antibody was performed. ASEM showed that signals derived from the DNA were localized around cells and in areas away from the cells (**Figures 6C–G**). These signals were aligned in a line, resembling fiber structures. The length of the strings varied, but strings exceeding 20 μm were readily observed. Such structures were not observed in the negative control samples that were labeled with normal mouse IgG primary antibody (**Figure 6H**). Thus, eDNA appeared to localize to the biofilm–surface interface, thereby contributing to biofilm formation by *P. acnes*.

## Discussion

*Propionibacterium acnes* has been reported to cause infections associated with implanted medical aids, such as cardiac devices (Chua et al., 1998; Zedtwitz-Liebenstein et al., 2003; Haidar et al., 2010), breast implants (Del Pozo et al., 2009; Rieger et al., 2009), and prosthetic joints (Piper et al., 2009). Rohacek et al. (2010) conducted culture tests of 115 asymptomatic pacemakers using sonication, which led to the isolation of mainly *P. acnes* and CNS. During follow-up, two patients for whom CNS was detected developed an infection, whereas no infection was observed in patients with detectable *P. acnes* (Rohacek et al., 2010). The clinical importance of bacterial colonization on asymptomatic cardiac devices is still open to discussion, because the occurrence of infection in such cases remains low (Baddour, 2010; Rohacek et al., 2010). However, considering the increasing number of patients with cardiac devices and their long-term use, it is important to elucidate the role and mechanism of the bacterial colonization of these devices. For microbiological diagnosis of cardiac device infections, conventional pocket swabs and tissue cultures are useful; however, bacteria are not detected in up to 30% of such infections (Dy Chua et al., 2005; Viola et al., 2010). Sonication of fluid cultures of explanted cardiac devices is more sensitive than conventional swab cultures for detecting bacteria (Rohacek et al., 2010; Mason et al., 2011). Ultrasonic treatment is effective for improving sensitivity because it promotes detachment of bacteria adhering to the surface of the device. In this study, we demonstrated that a simple stamp culture method, whereby the pacemakers are pressed on the surface of the agar medium can be used to visually evaluate bacteria adhering to the surface of the device (Supplementary Figure S1). The advantage of this approach is its ease of operation and visual read-out. Similar to other culture methods, it is difficult to completely eliminate contamination from skin bacteria during the extraction process. Therefore, for pacemakers with a small number of colonies, the possibility of skin-derived contamination should be considered. In contrast, colonization on almost the entire surface (e.g., PM12, PM13, and PM17 in Supplementary Figure S1) might suggest that a biofilm had formed on the device surface.

Consistent with previous results reported by Holmberg et al. (2009), *in vitro* biofilm formation by all *P. acnes* isolates was enhanced upon addition of glucose to the growth medium (data not shown). Thus far, which ECM components are important for biofilm formation by *P. acnes* has not been clarified. In this study, to determine the functional contributions of each ECM component (DNA, protein, and poly-GlcNAc) to biofilm formation, we assessed the effect of DNase I, proteinase K, and dispersin B on the biofilm formation. The results indicated that DNA and proteins are involved in biofilm formation, and that they contribute differently to biofilm formation among different *P. acnes* isolates. It has been reported that dispersin B inhibits biofilm formation of *S. aureus* and *S. epidermidis* (Izano et al., 2008), but it showed no effect against all *P. acnes* isolates used in this study (**Figure 1B**). Therefore, although GlcNAc was detected by ECM, poly-GlcNAc seemed not to be involved in biofilm formation by *P. acnes*. Interestingly, the same CCs presented similar ECM component ratios and SDS–PAGE patterns (**Figures 2, 3**). Although more isolates should be investigated, these results suggest that obtaining information about ECM components may be useful for typing of *P*. *acnes*.

Lysis of a subpopulation of the bacteria is sufficient for production of ECM containing a wide variety of cytoplasmic components. Observation of the 3D structure of *P*. *acnes* biofilms by CLSM demonstrated differences in the 3D distribution and proportion of living cells and dead cells in biofilms with different enzymatic susceptibilities and ECM component ratios (**Figure 4**). Viable cells were rarely observed in the thin biofilms formed in the presence of DNase I, which may suggest that eDNA plays a role in incorporating living cells in biofilms of *P. acnes*. The mechanism by which lysis of *P. acnes* cells is induced in the biofilms is still unclear. Hydrolase and RlpA, which were identified in the ECM, might be involved in lysis because they possess domains characteristic of cell wall-degrading enzymes (Jorgenson et al., 2014). We also identified enolase, a cytoplasmic glycolytic enzyme, among the ECM proteins (**Table 3**). Recently, it has been suggested that some cytoplasmic proteins, including enolase, are released from the cytoplasm and are associated with the cell surface, serving as moonlighting ECM components (Huberts and van der Klei, 2010; Henderson and Martin, 2013; Yoshii et al., 2017).

Immuno-electron microscopy by ASEM demonstrated that DNA existed as a fibrous structure not only around the cells but also away from them. The importance of DNA as a structural component in *P. aeruginosa* biofilm was reported by Whitchurch et al. (2002). Recently, Barnes et al. (2012) demonstrated that during early biofilm formation, *Enterococcus faecalis* produced extracellular structures containing DNA independently of cell lysis. In that study, immuno-scanning electron microscopy and fluorescent techniques revealed eDNA localization within intercellular filamentous structures and the septum, suggesting the possibility of DNA secretion from metabolically active cells. Currently, there is no conclusive evidence on whether *P. acnes* eDNA is secreted from living or dead cells, or from both. However, as the observed fibrous structure containing *P. acnes* eDNA is similar to that reported in *E*. *faecalis* by Barnes et al. (2012), secretion of eDNA might occur in the initial stage of *P*. *acnes* biofilm formation independently of cell lysis. In support of this hypothesis, the ECM of the JK12.2 biofilm contained more than three times the amount of DNA than the JK17.1 biofilm (**Figure 2A**), even though the proportion of dead cells of the JK12.2 biofilm was much smaller than that of the JK17.1 biofilm (**Figures 4A,B**).

This study revealed a part of the biofilm formation mechanism in *P. acnes* that has not been clarified in detail thus far. These findings could provide an important clue to understand the mechanism of device colonization and to control biofilm-associated infection caused by *P. acnes*.

## Author Contributions

K-iO, RN, TI, KH, and YM conceived and designed the experiments. K-iO, SY, SS, MS, and CS performed the experiments. K-iO, SS, and CS analyzed the data. K-iO, RN, SS, CS, and YM wrote the paper.

## Conflict of Interest Statement

The authors declare that the research was conducted in the absence of any commercial or financial relationships that could be construed as a potential conflict of interest.

## Footnotes

1 http://grants.nih.gov/grants/guide/pa-files/PA-07-288.html

2 http://www.ncbi.nlm.nih.gov/BLAST/

3 https://pubmlst.org/pacnes/

4 https://pubmlst.org/pacnes/
