# Supplementary material for: The Composition and Structure of Biofilms Developed by Propionibacterium acnes Isolated from Cardiac Pacemaker Devices
Source: Front Microbiol. 2018 Feb 14;9:182. doi: 10.3389/fmicb.2018.00182 (PMC5817082; doi:10.3389/fmicb.2018.00182)
Supplement: Supplementary file 1 [file Presentation_1.PDF]

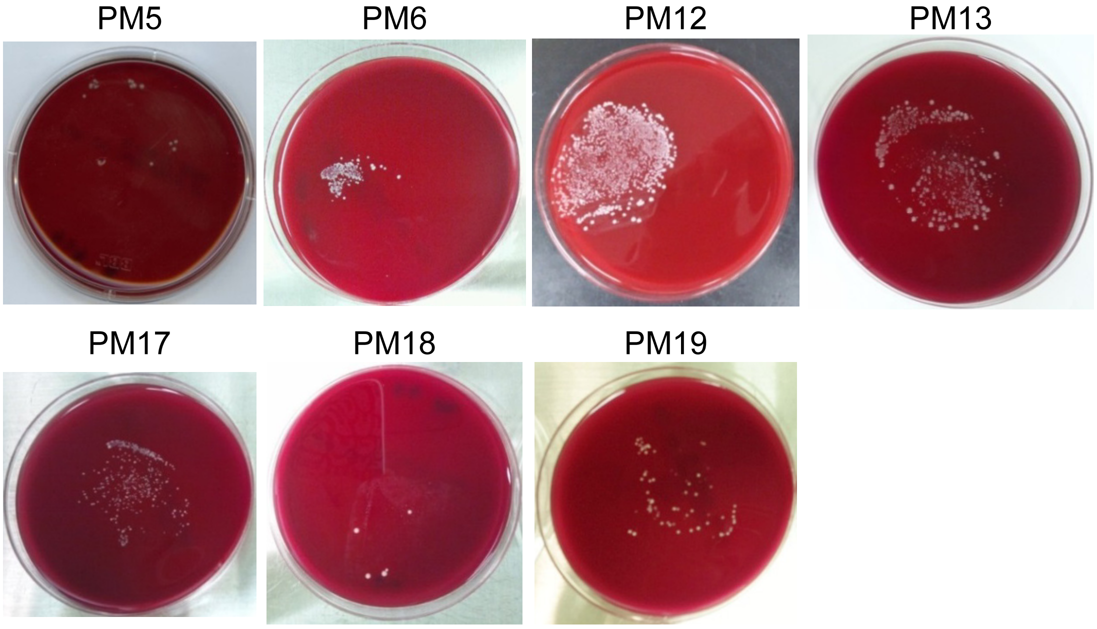

FIG S1 Culture tests of cardiac pacemaker devices.
Photographs showing culture-positive blood-agar plates on which the removed pacemakers were stamped and subsequently incubated anaerobically at 37ºC for 7 days. The pacemaker numbers are shown on the photographs.

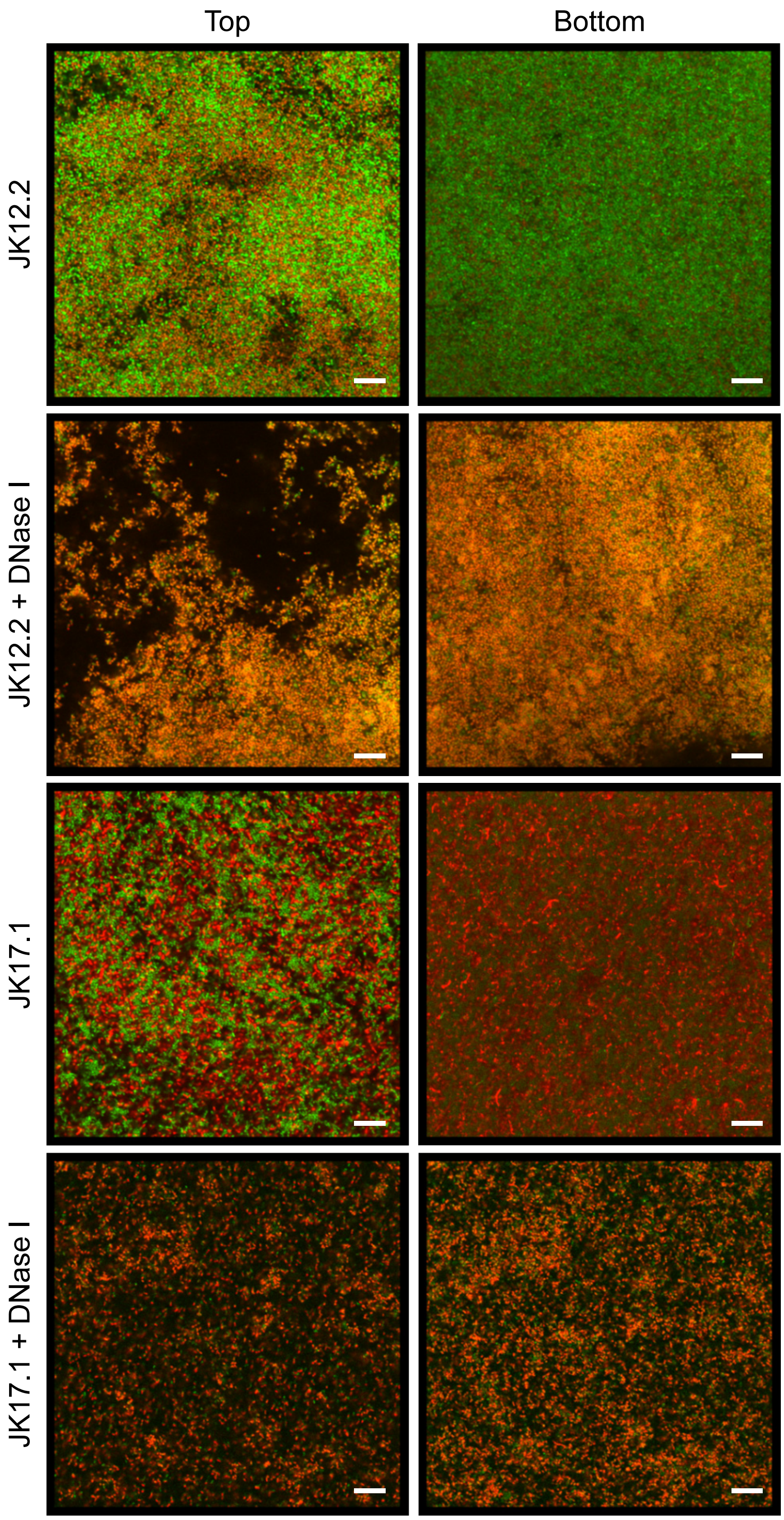

FIG S2 Live/dead imaging of *P. acnes* biofilms.
Two-dimensional images of Fig. 4. Living and dead cells were stained by SYTO9 (green) and propidium iodide (red), respectively. Top-side and bottom-side views are shown. Bars represent 10 µm.

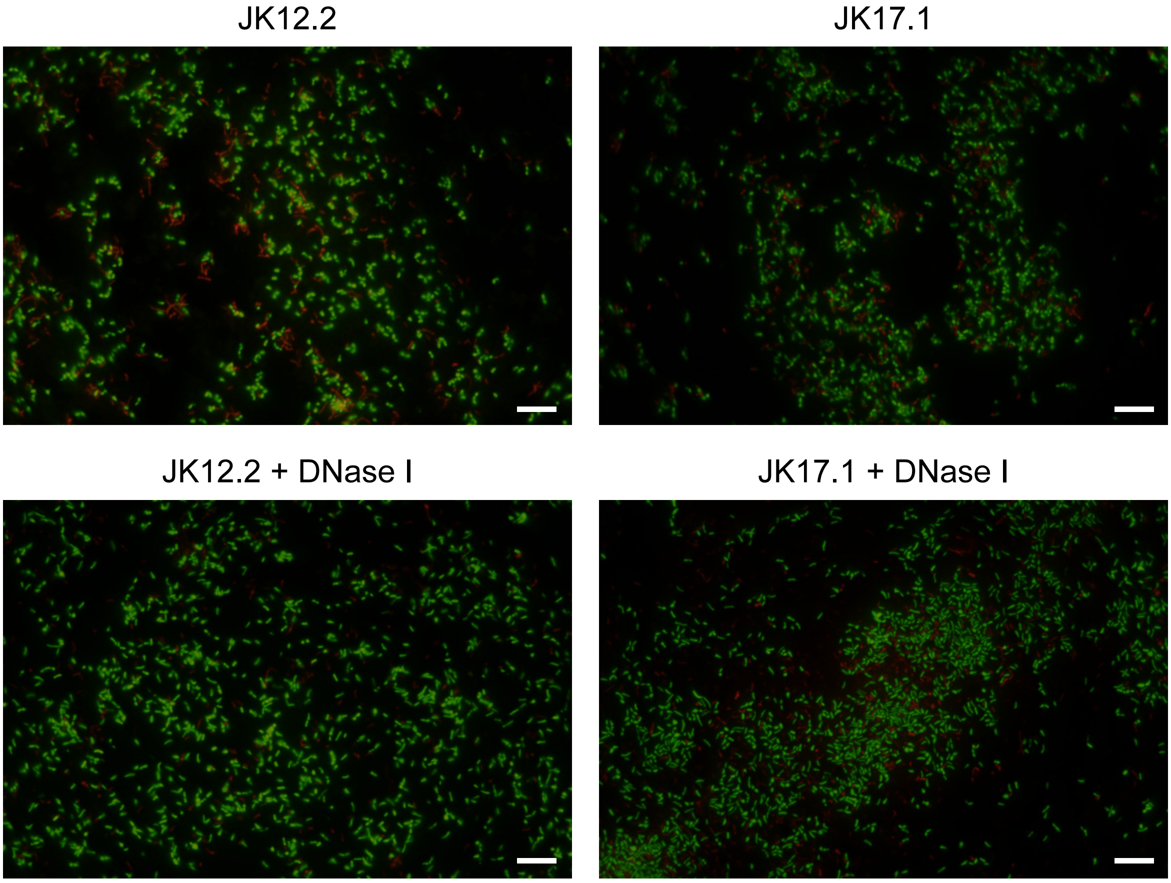

FIG S3 Live/dead imaging of planktonic cells of *P. acnes* cultured in the biofilm condition. Planktonic cells were collected from biofilm cultures by centrifugation, and subsequently living and dead cells were stained by SYTO9 (green) and propidium iodide (red), respectively. Fluorescence microscopy images are shown. Bars represent 10 µm.
